# Supplementary material for: Endothelial SMAD1/5 signaling couples angiogenesis to osteogenesis in juvenile bone
Source: Commun Biol. 2024 Mar 13;7:315. doi: 10.1038/s42003-024-05915-1 (PMC10937971; doi:10.1038/s42003-024-05915-1)
Supplement: Supplementary file 2 — Supplementary Information [file 42003_2024_5915_MOESM2_ESM.pdf]

SUPPLEMENTARY INFORMATION

Endothelial SMAD1/5 signaling couples angiogenesis to osteogenesis in juvenile bone

Authors

Annemarie Lang, Andreas Benn, Joseph Collins, Angelique Wolter, Tim Balcaen, Greet Kerckhofs, An Zwijsen, Joel D. Boerckel

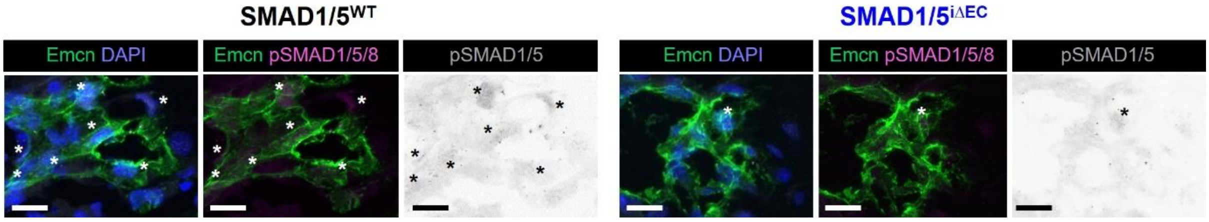

Supplementary Figure 1. ***Reduction of endothelial phosphoSMAD1/5/8-positive ECs in the bone marrow of SMAD1/5$^{i\Delta EC}$ mice.*** Mice were injected postnatal day 19-21 (P19-21) and samples were collected at P28. Figures show representative images of phospho(p)SMAD1/5/8 staining in EMCN positive endothelial cells in the diaphysis (P28; representative for n^{WT}= 4; $n^{i\Delta EC}$= 6). Scale bars indicate 20 µm. Asterisks indicate positive staining.

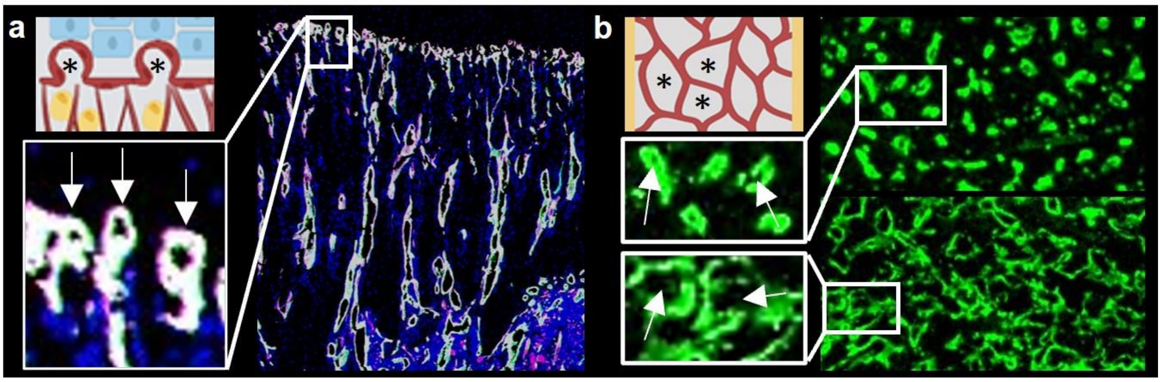

Supplementary Figure 2. Quantification of vascular arches and loops. (**a**) Vascular columns are linked by tubular arches next to the growth plate chondrocytes. (**b**) Vascular loops are defined by a closed enclosure lined with Emcn$^+$ cells around a lumen. * Asterisk in schematics indicate exemplary representation of either arches (a) or loops (b). Arrows indicate either exemplary arches (a) or loops (b) in magnifications.

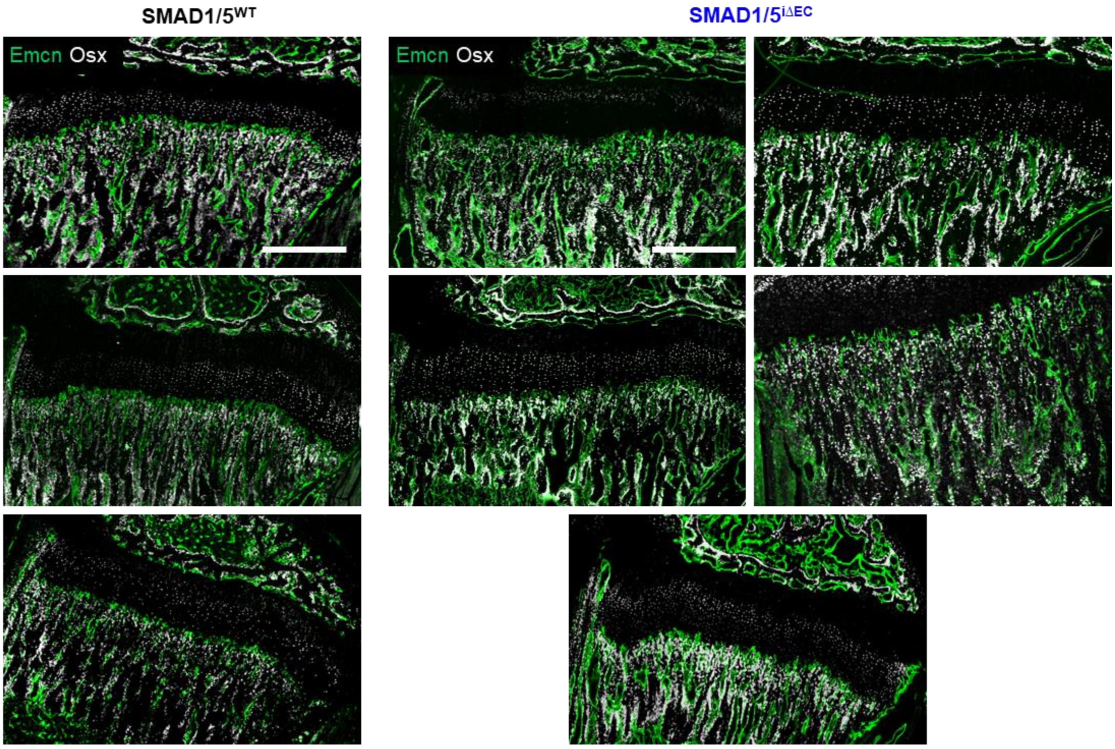

Supplementary Figure 3. ***Additional images on co-localization of active osteoprogenitors in the metaphyseal area*****.** All images of EMCN and OSX staining are in the tibial metaphysis (P28; n^{WT}= 3; $n^{i\Delta EC}$= 5). Scale bars indicate 500 µm.

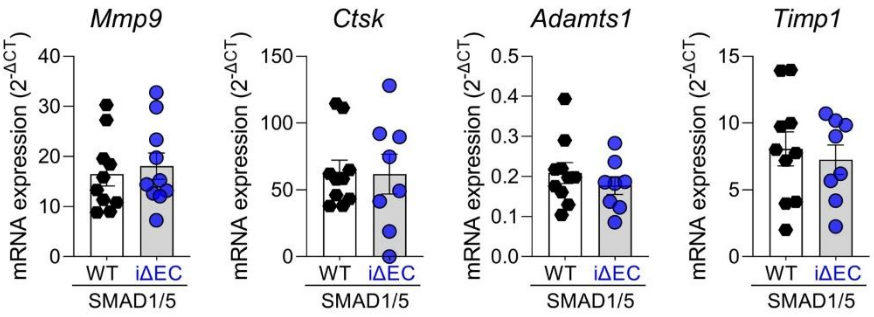

Supplementary Figure 4. Relative mRNA expression analysis of *Mmp9, Ctsk, Adamts1* and *Timp1* normalized to *Hprt* in the epi-/metaphysis. (P28; n= 10).

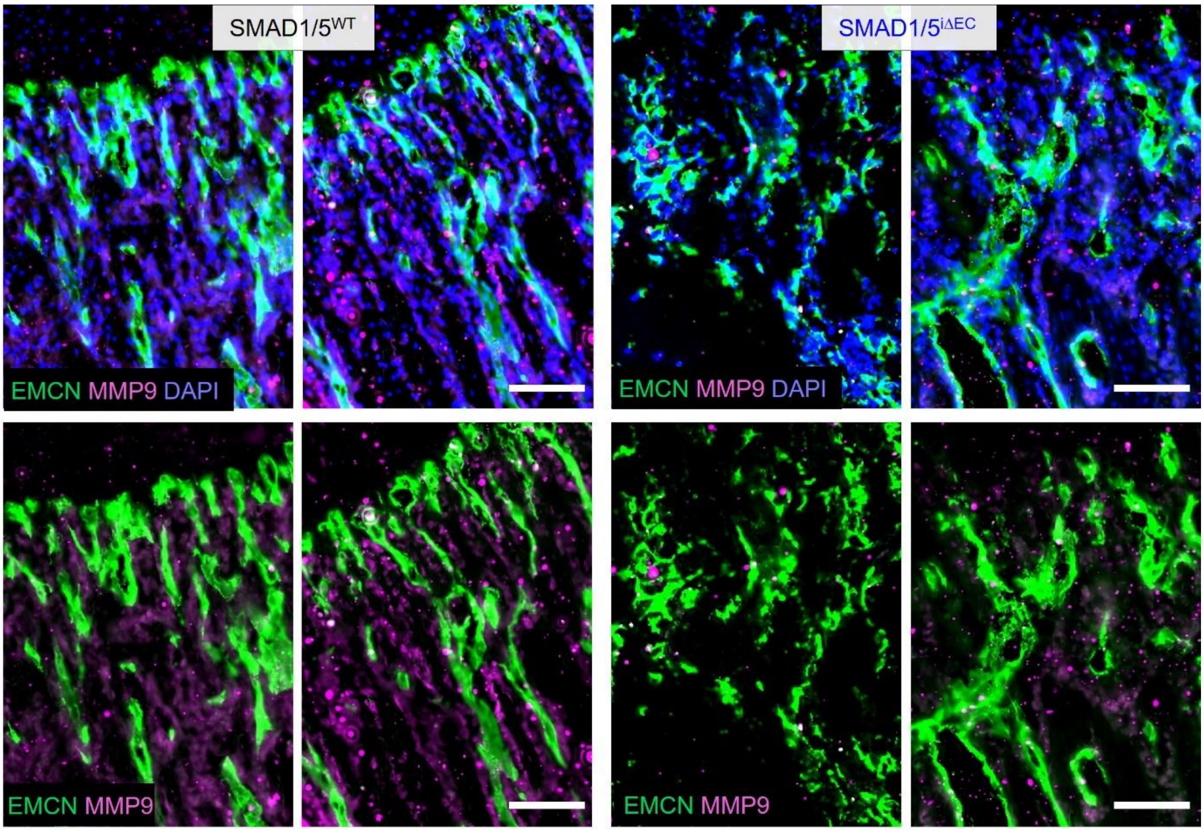

Supplementary Figure 5. MMP9 and EMCN staining in the metaphysis at 14 days post-tamoxifen injection (P35). Representative images displaying two independent samples (n= 2) per group. Scale bars indicate 100 µm.

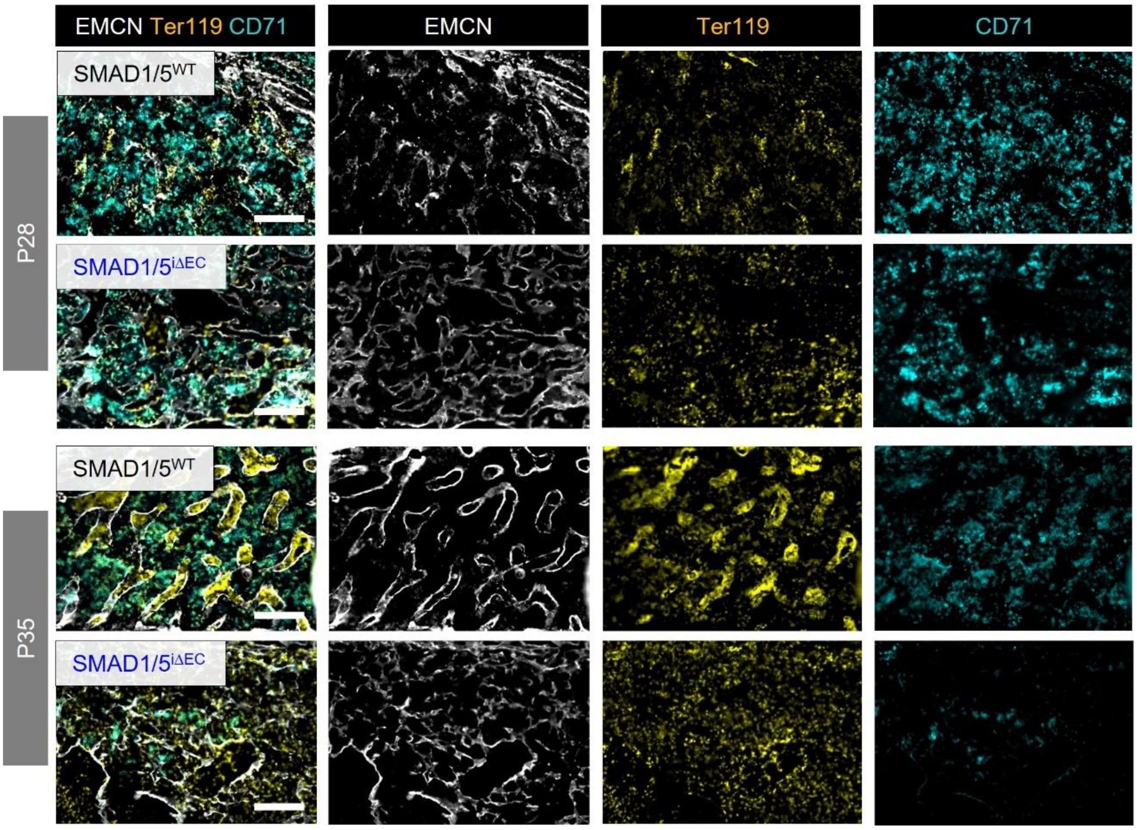

Supplementary Figure 6. EMCN, Ter119 and CD71 staining in the diaphysis. Representative images for n= 2-7 per group. Scale bars indicate 250 µm.

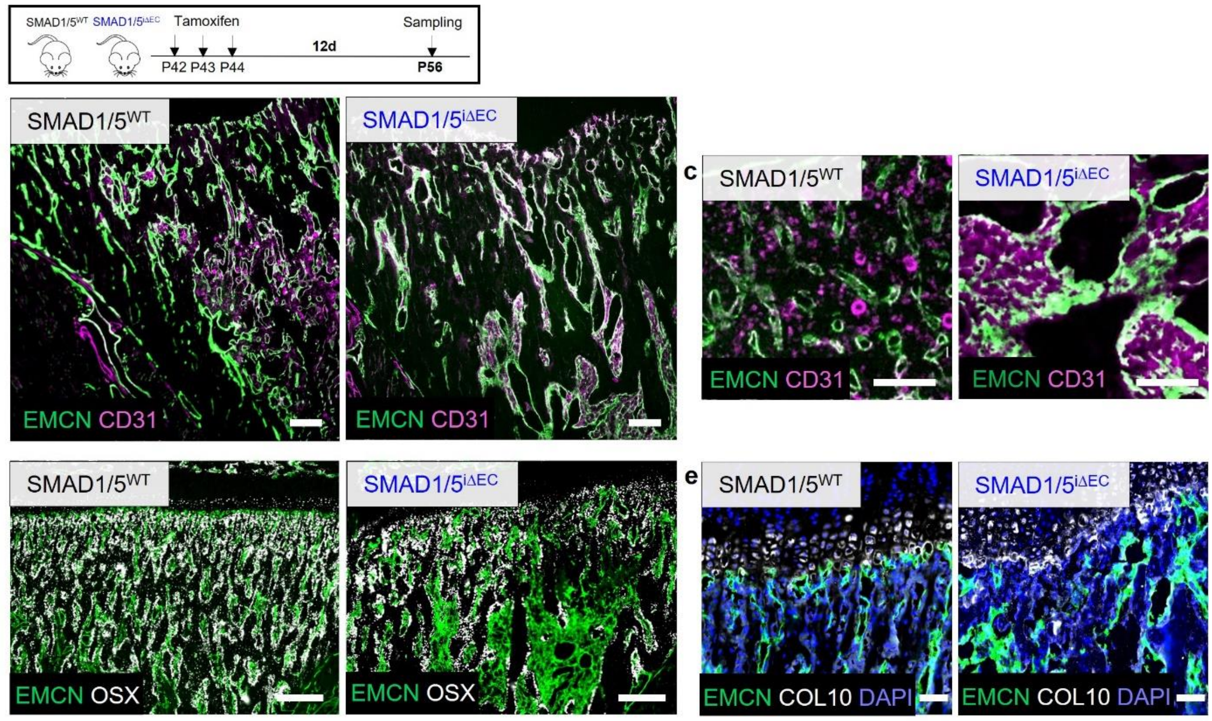

Supplementary Figure 7. Endothelial SMAD1/5 maintains morphology and function of metaphyseal and diaphyseal capillaries during early adolescent. (**a**) Tamoxifen treatment and long term sampling scheme. Mice were injected postnatal day 42-44 (P42-44) and samples were collected at P56 (8 weeks - 5 weeks post-weaning). Representative images of (**b**) EMCN and CD31 staining in the metaphysis or (**c**) diaphysis (P56; n^{WT}= 3; $n^{i\Delta EC}$= 2). Representative images of (**d**) EMCN and OSX or (**e**) EMCN, Col X and DAPI staining in the metaphysis (P56; n^{WT}= 3; $n^{i\Delta EC}$= 2). All scale bars indicate 250 µm (**b**, **d**), 125 µm (**c**) or 62.5 µm (**e**).

Supplementary Table 1: Primer sequences

**Genes**	**Gene Primer Sequence (5’ to 3’)**	
	**Forward**	**Reverse**
Emcn	CAGTGAAGCCACTGAGACCA	ACGTCACTTTTGGTCGTTCC
Id1	GCTCTACGACATGAACGGCT	CTGGAACACATGCCGCCT
Sp7	ACCAGAAGCGACCACTTGAG	TAGGGGAACAGAGAGAGCCC
Mmp9	CGACTTTTGTGGTCTTCCCCA	TCCCACTTGAGGCCTTTGAA
Ctsk	CAGTGTTGGTGGTGGGCTAT	CATGTTGGTAATGCCGCAGG
Adamts1	GTTCCACATCCTGAGGCGAA	TGGTTTCCACATAACGGGGG
Timp1	TGGGTTCCCCAGAAATCAACG	GCTTTCCATGACTGGGGTGT
Hprt	GTTGGGCTTACCTCACTGCT	TAATCACGACGCTGGGACTG
