# Supplementary material for: Endothelial SMAD1/5 signaling couples angiogenesis to osteogenesis in juvenile bone
Source: Commun Biol. 2024 Mar 13;7:315. doi: 10.1038/s42003-024-05915-1 (PMC10937971; doi:10.1038/s42003-024-05915-1)
Supplement: Supplementary file 3 — Description of additional supplementary files [file 42003_2024_5915_MOESM3_ESM.docx]

Description of Additional Supplementary Files

**File name:** Supplementary Data 1

**Description:** The source data behind the graphs and charts in the paper
