# Supplementary material for: Endothelial SMAD1/5 signaling couples angiogenesis to osteogenesis in juvenile bone
Source: Commun Biol. 2024 Mar 13;7:315. doi: 10.1038/s42003-024-05915-1 (PMC10937971; doi:10.1038/s42003-024-05915-1)
Supplement: Supplementary file 5 — Reporting summary [file 42003_2024_5915_MOESM5_ESM.pdf]

nature portfolio

Corresponding author(s): Tamer Önder

Last updated by author(s): Feb 13, 2024

Reporting Summary

Nature Portfolio wishes to improve the reproducibility of the work that we publish. This form provides structure for consistency and transparency in reporting. For further information on Nature Portfolio policies, see our Editorial Policies and the Editorial Policy Checklist.

Statistics

For all statistical analyses, confirm that the following items are present in the figure legend, table legend, main text, or Methods section.

n/a	Confirmed	
☐	☒	The exact sample size (*n*) for each experimental group/condition, given as a discrete number and unit of measurement
☐	☒	A statement on whether measurements were taken from distinct samples or whether the same sample was measured repeatedly
☐	☒	The statistical test(s) used AND whether they are one- or two-sided<br>*Only common tests should be described solely by name; describe more complex techniques in the Methods section.*
☒	☐	A description of all covariates tested
☒	☐	A description of any assumptions or corrections, such as tests of normality and adjustment for multiple comparisons
☐	☒	A full description of the statistical parameters including central tendency (e.g. means) or other basic estimates (e.g. regression coefficient) AND variation (e.g. standard deviation) or associated estimates of uncertainty (e.g. confidence intervals)
☐	☒	For null hypothesis testing, the test statistic (e.g. *F*, *t*, *r*) with confidence intervals, effect sizes, degrees of freedom and *P* value noted<br>*Give P values as exact values whenever suitable.*
☒	☐	For Bayesian analysis, information on the choice of priors and Markov chain Monte Carlo settings
☒	☐	For hierarchical and complex designs, identification of the appropriate level for tests and full reporting of outcomes
☒	☐	Estimates of effect sizes (e.g. Cohen's *d*, Pearson's *r*), indicating how they were calculated

Our web collection on statistics for biologists contains articles on many of the points above.

Software and code

Policy information about availability of computer code

Data collection	Colony quantification was performed using ImageJ (Rasband, W.S., ImageJ, U. S. National Institutes of Health, Bethesda, Maryland, USA, https://imagej.nih.gov/ij/, 1997-2016.). qRT-PCR results were analyzed by Microsoft Excel. The flow cytometry data were collected with BD Accuri C6 software.
Data analysis	GSEA-4.3.2, R-4.3.2, Microsoft Excel, ImageJ, MAGeCK, GraphPad Prism 10

For manuscripts utilizing custom algorithms or software that are central to the research but not yet described in published literature, software must be made available to editors and reviewers. We strongly encourage code deposition in a community repository (e.g. GitHub). See the Nature Portfolio guidelines for submitting code & software for further information.

Data

Policy information about availability of data

All manuscripts must include a data availability statement. This statement should provide the following information, where applicable:

- Accession codes, unique identifiers, or web links for publicly available datasets
- A description of any restrictions on data availability
- For clinical datasets or third party data, please ensure that the statement adheres to our policy

RNA-sequencing data are deposited to the NCBI GEO database with the accession number GSE225151.

Research involving human participants, their data, or biological material

Policy information about studies with human participants or human data. See also policy information about sex, gender (identity/presentation), and sexual orientation and race, ethnicity and racism.

Reporting on sex and gender	*Use the terms sex (biological attribute) and gender (shaped by social and cultural circumstances) carefully in order to avoid confusing both terms. Indicate if findings apply to only one sex or gender; describe whether sex and gender were considered in study design; whether sex and/or gender was determined based on self-reporting or assigned and methods used. Provide in the source data disaggregated sex and gender data, where this information has been collected, and if consent has been obtained for sharing of individual-level data; provide overall numbers in this Reporting Summary. Please state if this information has not been collected. Report sex- and gender-based analyses where performed, justify reasons for lack of sex- and gender-based analysis.*
Reporting on race, ethnicity, or other socially relevant groupings	*Please specify the socially constructed or socially relevant categorization variable(s) used in your manuscript and explain why they were used. Please note that such variables should not be used as proxies for other socially constructed/relevant variables (for example, race or ethnicity should not be used as a proxy for socioeconomic status). Provide clear definitions of the relevant terms used, how they were provided (by the participants/respondents, the researchers, or third parties), and the method(s) used to classify people into the different categories (e.g. self-report, census or administrative data, social media data, etc.) Please provide details about how you controlled for confounding variables in your analyses.*
Population characteristics	*Describe the covariate-relevant population characteristics of the human research participants (e.g. age, genotypic information, past and current diagnosis and treatment categories). If you filled out the behavioural & social sciences study design questions and have nothing to add here, write "See above."*
Recruitment	*Describe how participants were recruited. Outline any potential self-selection bias or other biases that may be present and how these are likely to impact results.*
Ethics oversight	iPSC generation was carried out under a protocol approved by Koç University IRB.

Note that full information on the approval of the study protocol must also be provided in the manuscript.

Field-specific reporting

Please select the one below that is the best fit for your research. If you are not sure, read the appropriate sections before making your selection.

☒ Life sciences ☐ Behavioural & social sciences ☐ Ecological, evolutionary & environmental sciences

For a reference copy of the document with all sections, see nature.com/documents/nr-reporting-summary-flat.pdf

Life sciences study design

All studies must disclose on these points even when the disclosure is negative.

Sample size	No sample size calculation was performed. Exact number of technical and biological repeats are listed in figure legends.
Data exclusions	No data were excluded.
Replication	We performed 2 independent repliates for reprogramming screen and at least 3 replicates for all other reprogramming assays and at least two replicates were performed for other assays such as RT-qPCR and western blot. All results were reproducible.
Randomization	We did not perform randomization as this study does not allocation of samples into experimental groups.
Blinding	We were not blind to the experimental analysis. Results were confirmed at least by 2 authors independently.

Reporting for specific materials, systems and methods

We require information from authors about some types of materials, experimental systems and methods used in many studies. Here, indicate whether each material, system or method listed is relevant to your study. If you are not sure if a list item applies to your research, read the appropriate section before selecting a response.

Materials & experimental systems

n/a	Involved in the study	
☐	☒	Antibodies
☐	☒	Eukaryotic cell lines
☒	☐	Palaeontology and archaeology
☐	☒	Animals and other organisms
☒	☐	Clinical data
☒	☐	Dual use research of concern
☒	☐	Plants

Methods

n/a	Involved in the study	
☒	☐	ChIP-seq
☐	☒	Flow cytometry
☒	☐	MRI-based neuroimaging

Antibodies

Antibodies used	USP22 (NOVUS, cat. # NBP1-49644), Ubiquityl-Histone H2A(Lys119) (Cell Signaling, cat. # 8240S), Ubiquityl-Histone H2B (Lys120)(Cell Signaling, cat. # 5546S), H2B (Abcam, cat. #ab52599-1:20000), Tubulin and GAPDH(Abcam, cat. #ab9485) at 1:1000. Secondary antibodies (Abcam, cat. # ab97051 or ab97023) at 1:5000.
Validation	USP22 antibody was validated in this study through overexpression and knockout of target gene by western blot.

Eukaryotic cell lines

Policy information about cell lines and Sex and Gender in Research

Cell line source(s)	Wild type iPSCs derived from a Caucasian female. dH1f cells were a gift from Dr. George Daley (Harvard Medical School, Boston MA), HEK293T cells were a gift from Dr.Robert Weinberg (Whitehead Institute, Boston MA).
Authentication	Immunofluorescence and RT-qPCR of cell-specific genes were performed to authenticate each cell line.
Mycoplasma contamination	All cell lines were tested negative for mycoplasma contamination.
Commonly misidentified lines (See ICLAC register)	*Name any commonly misidentified cell lines used in the study and provide a rationale for their use.*

Animals and other research organisms

Policy information about studies involving animals; ARRIVE guidelines recommended for reporting animal research, and Sex and Gender in Research

Laboratory animals	8-12 week old male SCID mice (obtained from Koc University Animal Research Facility) were used for teratoma formation assay.
Wild animals	The study did not involve wild animals.
Reporting on sex	This information was not collected.
Field-collected samples	The study did not involve samples collected in the field.
Ethics oversight	All experiments were carried out under a protocol approved by Koç University Animal Experiments Ethics Committee.

Note that full information on the approval of the study protocol must also be provided in the manuscript.

Plants

Seed stocks	*Report on the source of all seed stocks or other plant material used. If applicable, state the seed stock centre and catalogue number. If plant specimens were collected from the field, describe the collection location, date and sampling procedures.*
Novel plant genotypes	*Describe the methods by which all novel plant genotypes were produced. This includes those generated by transgenic approaches, gene editing, chemical/radiation-based mutagenesis and hybridization. For transgenic lines, describe the transformation method, the number of independent lines analyzed and the generation upon which experiments were performed. For gene-edited lines, describe the editor used, the endogenous sequence targeted for editing, the targeting guide RNA sequence (if applicable) and how the editor was applied.*
Authentication	*Describe any authentication procedures for each seed stock used or novel genotype generated. Describe any experiments used to assess the effect of a mutation and, where applicable, how potential secondary effects (e.g. second site T-DNA insertions, mosiacism, off-target gene editing) were examined.*

Flow Cytometry

Plots

Confirm that:

- ☐ The axis labels state the marker and fluorochrome used (e.g. CD4-FITC).
- ☐ The axis scales are clearly visible. Include numbers along axes only for bottom left plot of group (a 'group' is an analysis of identical markers).
- ☐ All plots are contour plots with outliers or pseudocolor plots.
- ☒ A numerical value for number of cells or percentage (with statistics) is provided.

Methodology

Sample preparation	Cell pellets were resuspended in 1% FBS in PBS (washing buffer). Centrifuged at 1500 rpm for 5 minutes at 4C. Cell pellets were washed once again and resuspended in 0.125 uL TRA-1-60 ab containing washing buffer and incubated for 1 hour on ice in dark. Cells were washed twice and resuspended in wash buffer for cytometry analysis.
Instrument	BD Accuri C6 was used.
Software	During data collection software of BD Accuri C6 was used. Analysis was performed on Microsoft Excel.
Cell population abundance	Cell populations were around 50% of all counts. 10000 cells were counted to ensure robust results.
Gating strategy	Negative control cells (without OSKM) and positive control cells (iPSCs) were used to define TRA-1-60 positive and negative cell boundaries.

- ☐ Tick this box to confirm that a figure exemplifying the gating strategy is provided in the Supplementary Information.
